# Silencing the nosocomial pathogen *Serratia marcescens* by glyceryl trinitrate

**DOI:** 10.4314/ahs.v18i1.2

**Published:** 2018-03

**Authors:** Hisham A Abbas, Ahmed M Elsherbini

**Affiliations:** 1 Department of Microbiology and Immunology, Faculty of Pharmacy, Zagazig University- Zagazig- Egypt; 2 Health Sciences College-Umm Al Qura University, AlQunfudah, Saudi Arabia

**Keywords:** *Serratia marcescens*, quorum sensing, virulence, glyceryl trinitrate

## Abstract

**Background:**

Quorum sensing is a cell-to-cell communication system in bacteria that controls the production of virulence factors. *Serratia marcescens* is a causative agent of hospital-acquired infections that shows high resistance to antibiotics. This makes the treatment of these infections difficult. Quorum sensing regulates the production of virulence factors of *S. marcescens* such as prodigiosin, protease, swimming and swarming motilities and formation of biofilms. Inhibition of quorum sensing may be an alternative to antibiotic treatment to avoid emergence of resistance.

**Objectives:**

Testing the ability of glyceryl trinitrate to inhibit quorum sensing and virulence factors of *Serratia marcescens*.

**Methods:**

The inhibiting activities of sub-inhibitory concentration of glyceryl trinitrate against the quorum-sensing regulated violacein pigment in *Chromobacterium violaceum* CV026 was performed to evaluate the anti-quorum sensing effect of glyceryl trinitrate. The anti-virulence activity was assessed against prodigiosin, protease, biofilm formation in addition to swimming and swarming motilities.

**Results:**

Glyceryl trinitrate at at a concentration of 0.25 mg/ml produced significant inhibitory effects against violacein (67.01%), prodigiosin (82.67%), protease, swimming (36.72%) and swarming (79.31%) motilities and biofilm formation (87.83%).

**Conclusion:**

Glyceryl trinitrate is a quorum sensing and virulence inhibitor that may be useful in treatment of nosocomial infections caused by *Serratia marcescens*.

## Introduction

*Serratia marcescens* is a nosocomial pathogen that can lead to many nosocomial infections such as those affecting urinary tract, respiratory tract in addition to wound infections. It has an arsenal of virulence factors that aid in escape from the immune system and causing these infections such as prodigiosin, proteases, biofilm and its ability to swim and swarm^1^–^3^. These virulence factors are controlled by a mechanism of intercellular communication termed quorum sensing. Quorum sensing enables bacterial cells to monitor their numbers and respond by coordinating the expression of genes including virulence genes^2^–^4^. Some strains of *Serratia marcescens* cause infections that are hard to treat. The underlying reason is their high resistance to many categories of antibiotics involving fluoquinolones, aminoglycosides and β-lactams^5^,^6^. The high virulence and resistance of *Serratia marcescens* necessitates seeking a new treatment strategy. It is supposed that quorum sensing inhibition is one useful approach that can help in treating infections without exerting stress on the growth of bacteria to avoid the evolution of antibiotic resistance. Furthermore, it can enhance the immune clearance of the pathogens^7^,^8^.

Glyceryl trinitrate (GTN) is an anti-hypertensive that has anti-microbial properties^9^,^10^. It could inhibit the growth of *Candida albicans* and *Pseudomonas aeruginosa* planktonic growth^10^,^11^. Moreover, GTN exerted antibiofilm activity against *Staphylococcus aureus, Staphylococcus epidermidis, Pseudomonas aeruginosa* and *candida albicans*^12^. In our previous study, GTN was found to inhibit quorum sensing in *Pseudomonas aeruginosa*^11^. GTN is an FDA approved drug that can be used topically for anal fissures at concentrations that can reach 0.4%^13^.

This study aimed to investigate the potential anti-quorum sensing and anti-virulence activities of GTN against the nosocomial pathogen *Serratia marcescens*.

## Materials and methods

### Media and chemicals

Tryptone soya broth, Mueller Hinton broth and Mueller Hinton agar were the products of Oxoid (Hampshire, UK). Luria-Bertani (LB) broth and LB agar were purchased from Lab M Limited (Lancashire, United Kingdom).Glyceryl trinitrate was obtained from POHL-Boskamp, Gmbh&Co., Hohenlockstedt, Germany (Stock solution of 1 mg/ml). Other chemicals were of pharmaceutical grade.

### Bacterial Strains

The *Serratia marcescens* isolate in this study was used in a previous study^14^. It is a clinical one obtained from an Intensive Care Unit patient admitted to Zagazig University Hospital by endotracheal aspiration. The MALDI-TOFF apparatus at the Clinical Pathology Department, Faculty of Medicine, Zagazig University was used for identification of this isolate. The biosensor strain *Chromobacterium violaceum* CV026 was obtained from the Department of Microbiology, Faculty of Pharmacy, Ain Shams University.

### Determination of Minimum Inhibitory Concentration (MIC)

The agar dilution method was used in determination of the minimum inhibitory concentration of GTN according to the Clinical Laboratory and Standards Institute Guidelines (CLSI)^15^. The tested strain was incubated overnight in tryptone soya broth (TSB) and the suspension was diluted with Mueller-Hinton broth in order to prepare a suspension with a turbidity approximating that of 0.5McFarland Standard. The suspension was further diluted with sterile saline (1:10). By using a micropipette, a standardized inoculum (approximately 10^4^ CFU per spot) was spotted on the surface of Mueller-Hinton agar plates containing different GTN concentrations and control plate without GTN. The MIC of GTN was the lowest concentration that inhibits growth on the plate after incubation at 37°C for 20 h.

### Effect of GTN on bacterial growth

The effect of sub-inhibitory concentration of GTN on the growth of the tested strain of *S. marcescens* was detected according to Nalca et al.^16^ overnight culture from *S. marcescens* was prepared in LB broth and adjusted to 0.5 McFarland Standard. The prepared suspension was used to inoculate LB broth containing 0.25 mg/ml of GTN and control LB broth without GTN so that the final inoculum is approximately 1×10^6^ CFU/ml. After overnight incubation at 37 °C, the optical densities of both cultures were measured at 600 nm by using Biotek Spectrofluorimeter (Biotek, USA).

### *Violacein* inhibition assay

To determine the quorum sensing inhibiting activity of GTN, the biosensor strain *Chromobacterium violaceum* CV026 was used according to Choo et al^17^. A suspension of the tested strain with OD_600_ of 1 was prepared from overnight culture. Aliquots of 100 µl of bacterial suspension were added to the wells of a 96-well microtiter plate to which aliquots of 100 µl of LB broth with N-hexanoyl homoserinelactone in the presence and absence of 0.25 mg/ml of GTN were delivered. The plate was incubated at 28°C for 16 h and was dried at 60°C. The purple pigment *violacein* was extracted by addition of aliquots of 100 µl of DMSO to the wells and incubation at 30°C with shaking. DMSO was used as negative control and the absorbance was measured at 590 nm using Biotek Spectrofluorimeter (Biotek, USA).

### Biofilm inhibition assay

The tested strain was reported as a strong biofilm forming isolate^14^. To determine the ability of GTN to inhibit biofilm formation, the modified method of Abraham et al.^18^ was used. A suspension of *S. marcescens* strain was prepared from overnight culture in tryptone soya broth (TSB) and its optical density was adjusted to OD_600_ of 0.4 (1×10^8^ CFU/ ml) was added. Aliquot of 10µl of the suspension was added to 1 ml amounts of fresh TSB with and without 0.25 mg/ml of GTN. Aliquots of 100 µl of TSB with and without GTN were delivered into the wells of 96 wells microtiter plate and incubated at 28 °C for 24 h. The planktonic cells were aspirated and the wells were washed three times with distilled water and left to dry. The attached cells were fixed with methanol for 20 minutes and stained with crystal violet (1%) for 20 minutes. The wells were washed and the attached dye was eluted by 33% glacial acetic acid. The absorbance was measured at 590 nm using Biotek Spectrofluorimeter (Biotek, USA). The percentage of biofilm inhibition was calculated using the following formula

% of biofilm inhibition = [OD_600_ control- OD_600_ in presence of ambroxol]/ OD_600_ control

### Microscopic visualization of biofilm inhibition by the light microscope and scanning electron microscope

In order to analyze biofilm inhibition, the method of Sakar et al.^19^ was followed with some modification. The biofilm of the tested strain of *Serratia marcescens* was formed on glass cover slips placed in polystyrene petri plates in the presence and absence of 0.25 mg/ml of GTN. The plates were incubated for 24 h at 28°C, the cover slips were washed with water three times and stained with crystal violet (1%) for 20 minutes. The cover slips were examined after staining under the light microscope at a 400X magnification. For scanning electron microscopic (SEM) analysis, biofilms formed on glass cover slips were fixed by glutaraldehyde (2.5%) for 120 minutes. After washing with distilled water, the cover slips were dehydrated by increasing concentrations of ethanol (50%, 60%, 70%, 80%, 90% and 100%) for 30 seconds. The samples were gold coated after critical-point drying and examined under scanning electron microscope^20^.

### Swimming and swarming motilities assay

The ability of GTN to block the swimming and swarming motilities was detected according to Matsuyama et al.^21^ For swimming assay, LB agar plates containing 0.3% agar with and without 0.25 mg/ml GTN were prepared. Overnight culture of *S.marcescens* in LB broth was prepared and 5µl of the suspension was inoculated into the center of the plates. Swarming LB gar plates with 0.5% agar containing 0.25% mg/ml of GTN and control plates were point inoculated with 5µl of the prepared suspension. The plates were incubated at 28°C for 20 h. The zones of swimming and swarming were measured. The experiment was made in triplicates and the results were averaged.

### Prodigiosin inhibition assay

The production of prodigiosin by *S. marcescens* was quantified in the presence and absence of GTN. The tested strain was grown overnight in LB broth and adjusted to OD_600_ of 0.4 and inoculated in 2 ml fresh LB broth and incubated at 28°C for 18 h. The cells were collected by centrifugation at 13000 rpm for 10 minutes. To extract prodigiosin, acidified ethanol (4% 1M HCl in ethanol) was used. The absorbance was measured at 534 nm using Biotek Spectrofluorimeter (Biotek, USA) and the degree of inhibition was determined. The experiment was made in triplicate and the results were averaged^22^.

### Protease assay

To assay the inhibitory activity of GTN on protease production, the qualitative skim milk agar method was used^23^. Skim milk LB agar plates (5% skim milk) with and without GTN (0.25 mg/ml) were prepared. The plates were surface inoculated, each with 5 µl of overnight culture of *S. marcescens* in LB broth and incubated at 28°C for 20h. The diameter of the clear zones surrounding the growth were measured.

### Statistical analysis

The inhibitory activities of GTN on quorum sensing and virulence factors of *Serratia marcescens* were compared by t test, Graph Pad Prism 5. P values <0.05 were considered statistically significant.

## Results

### Determination of MIC

Glyceryl trinitrate could not inhibit the growth of *Serratia marcescens* isolate at 0.5 mg/ml which is the maximum available concentration (stock solution of GTN is of concentration of 1 mg/ml), so MIC is equal to or higher than 1 mg/ml. the concentration selected to test the anti-quorum sensing and anti-virulence activities of GTN is 0.25 mg/ml which is equivalent to ¼ MIC or less.

### Determination of the effect of GTN on bacterial growth

**Figure 1:**
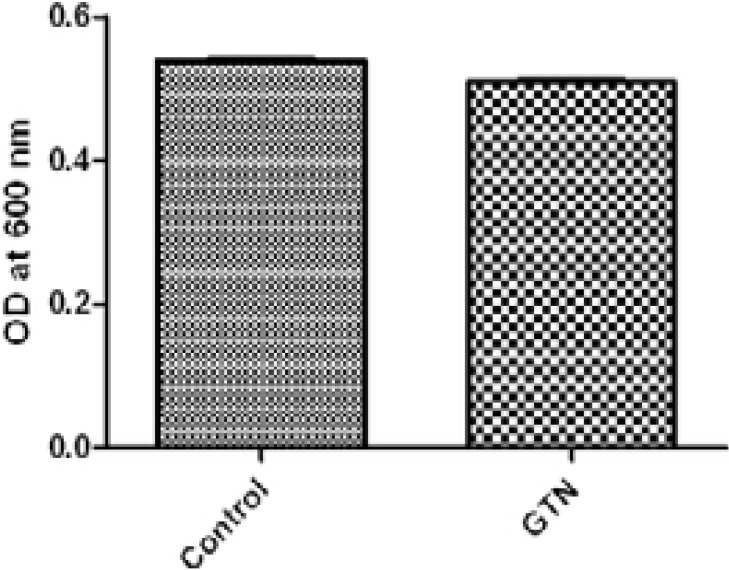
Effect of GTN on growth of *Serratia marcescens*. No statistically significant difference between OD_600_ of the GTN treated and untreated cultures after overnight incubation in LB broth.

To determine the effect of GTN on growth, the optical densities of the bacterial suspension at 600 nm following overnight incubation in LB broth were measured in the presence or absence of GTN. No statistically significant difference was found between the turbidities of the bacterial suspension with or without GTN indicating the lack of the effect of GTN on growth (Fig. 1).

### Assessment of biofilm inhibition

**Figure 2:**
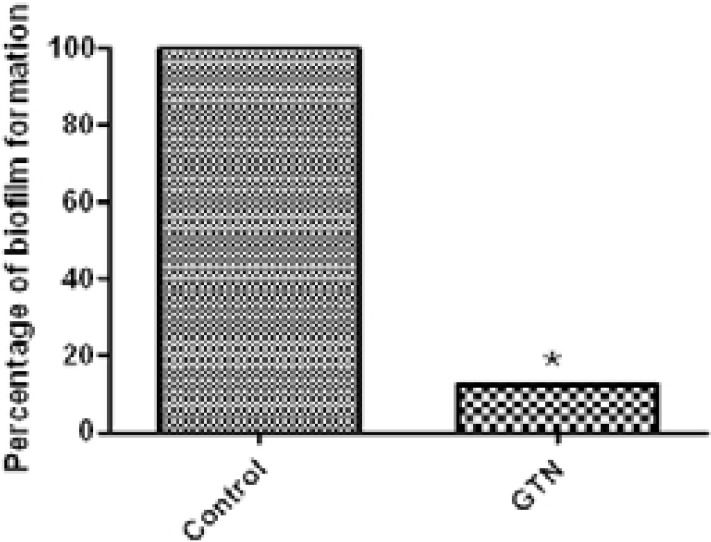
Biofilm inhibition of *S. marcescens* by GTN.*, significant P< 0.05.

The biofilm formation was quantified to show the ability of GTN to interfere with biofilm production. GTN at 0.25 mg/ml produced statistically significant reduction in biofilm biomass (p<0.05). The percentage of biofilm inhibition reached 87.83% (Fig. 2).

**Figure 3:**
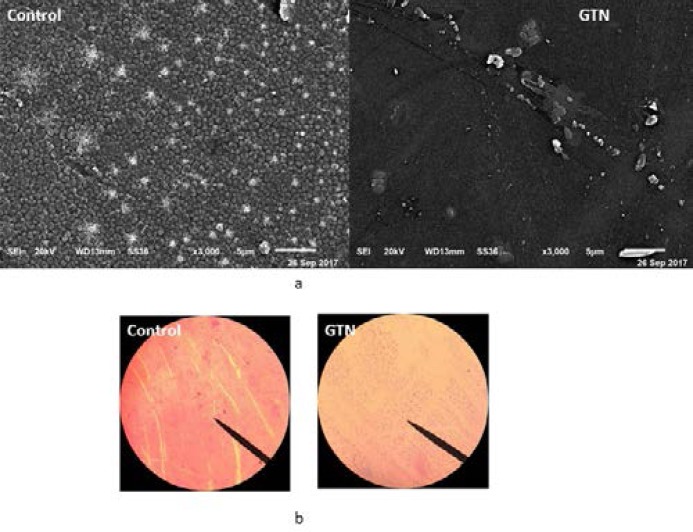
Microscopic analysis of biofilm inhibition by GTN (a) SEM images and (b) Light microscopic images of GTN treated and untreated biofilms.

To further explore the biofilm inhibiting activity of GTN, light microscopic examination of biofilms formed on glass cover slips in the presence and absence of GTN was performed. In the presence of GTN, both the thickness and surface coverage were markedly decreased (Fig. 3). Furthermore, the SEM images clearly illustrated the biofilm inhibiting activity of GTN. In GTN treated sample, very few scattered cells were observed as compared to the untreated sample that showed the complex biofilm structure with aggregated cells.

### Inhibition of violacein production

**Figure 4:**
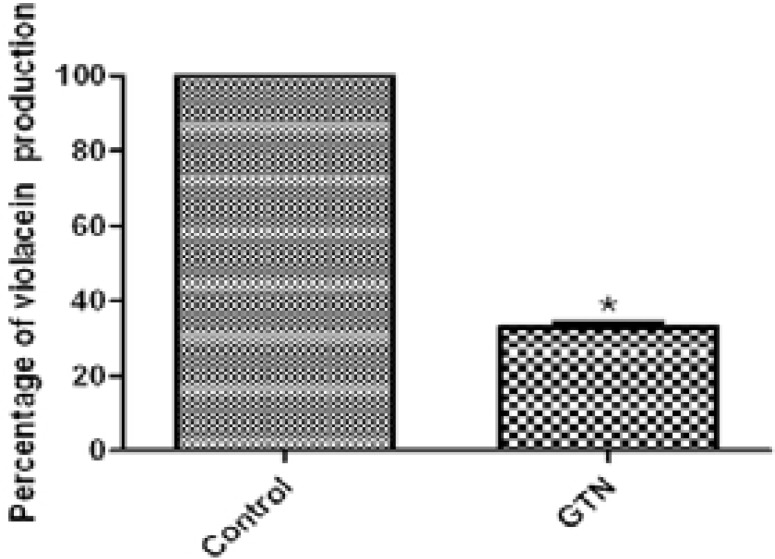
Inhibition of violacein pigment of Chromobacterium violaceum CV026 by GTN. *, significant P< 0.05.

To prove the quorum sensing inhibition by GTN, the production of *violacein* pigment in the biosensor strain *Chromobacterium violaceum* CV026 that is controlled by quorum sensing mechanism was monitored. GTN significantly diminished *violacein* production (67.01%). This confirms that GTN is a quorum sensing inhibitor (Fig. 4).

### Prodigiosin inhibition assay

**Figure 5:**
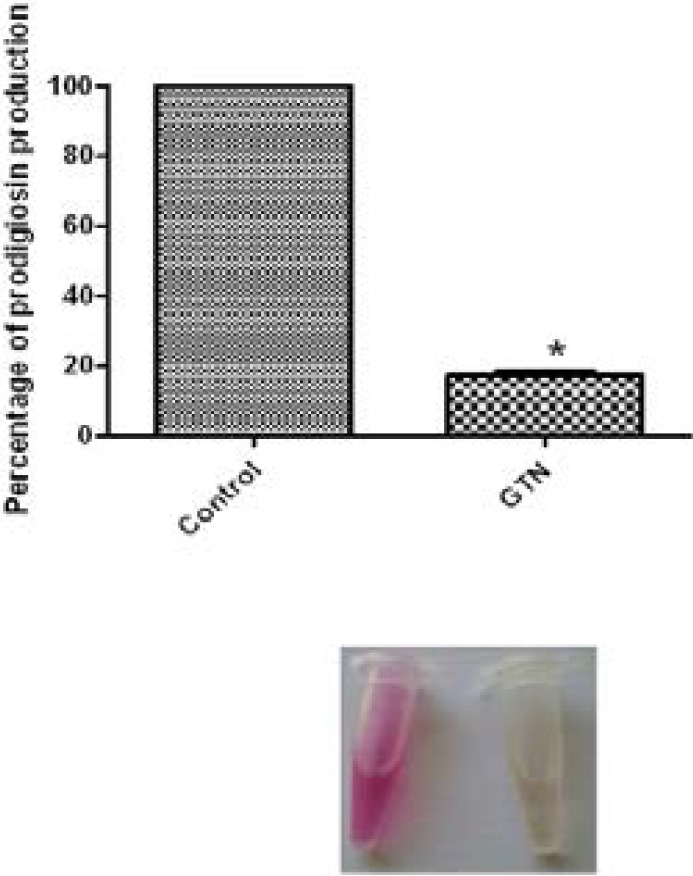
Inhibition of prodigiosin pigment of *Serratia marcescens* by GTN. *, significant P< 0.05.

Prodigiosin is another quorum-sensing controlled pigment that is produced by *S. marcescens*. GTN showed a significant ability to inhibit prodigiosin production. The inhibition percentage achieved was 82.67% (Fig 5).

### Inhibition of Protease Production

**Figure 6:**
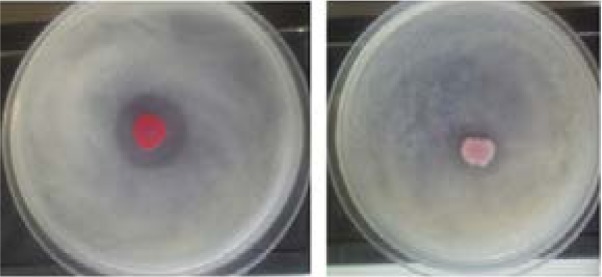
Inhibition of protease production by the skim milk agar method.

The skim milk agar method was used for the qualitative assay of protease inhibition. GTN could interfere with protease production as shown by decreasing the clear zone of proteolysis (Fig. 6).

### Inhibition of swimming and swarming

**Figure 7:**
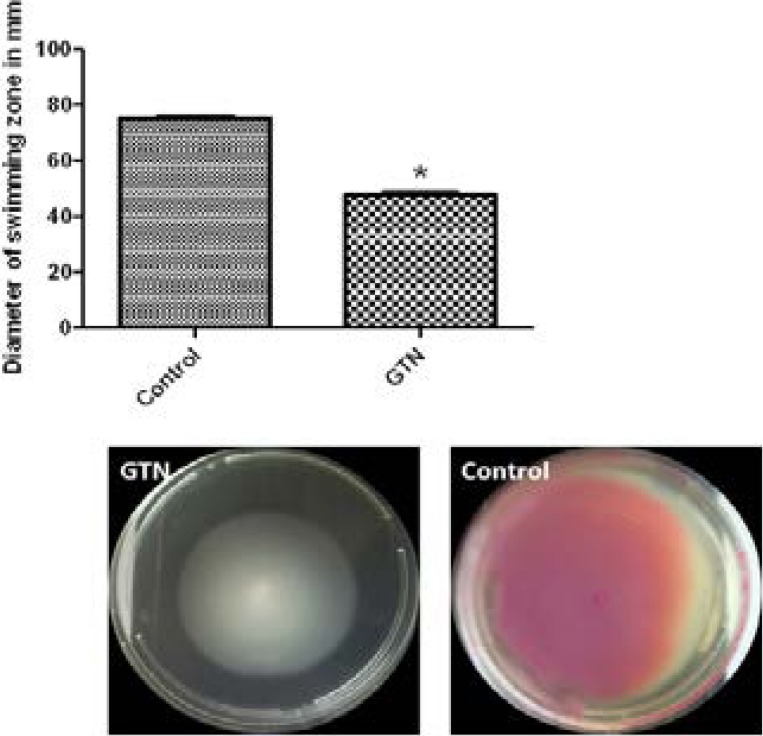
Inhibition of swimming motility by GTN. *, significant P< 0.05.

**Figure 8:**
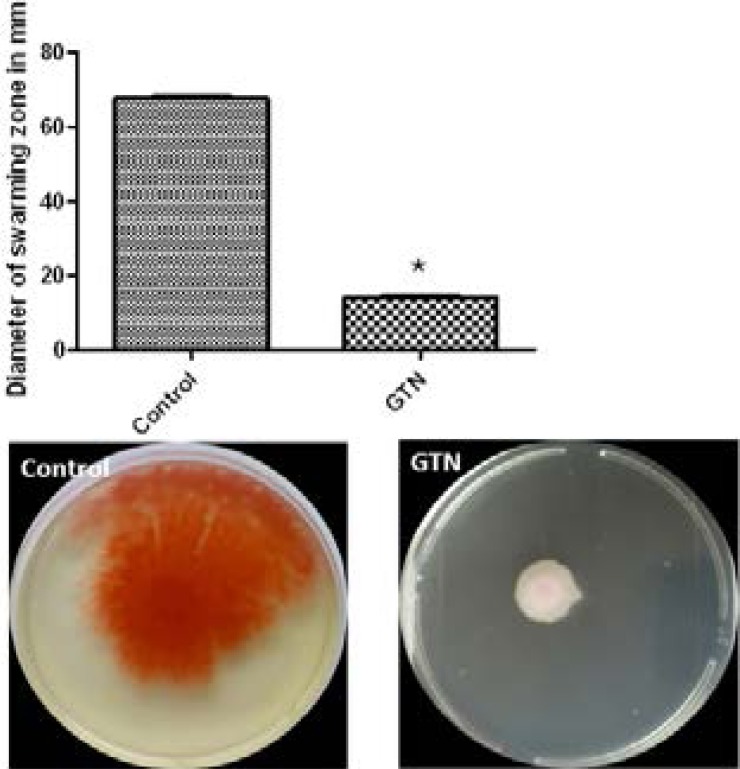
Inhibition of swarming motility by GTN. *, significant P< 0.05.

Swimming and swarming motility are important for adhesion and biofilm formation. In the presence of GTN, swimming motility was reduced by 36.72%, while swarming motility was decreased to the level of 79.31% (Fig. 7&8).

## Discussion

*Serratia marcescens* is a nosocomial bacterium that is considered the seventh most common pathogen that causes nosocomial pneumonia and the tenth most common one that is responsible for hospital acquired blood stream infections^24^.

This study was performed to detect the possible quorumsensing mediated virulence factors inhibition by sub-inhibitory concentration of glyceryl trinitrate in *Serratia marcescens*.

The underlying reason for targeting quorum sensing is to bypass the stress on bacterial growth that leads to emergence of resistance and finding another way to treat infection in the light of the high antibiotic resistance of *Serratia marcescens*^25^. Quorum sensing is a key regulator of the pathogenicity in many bacteria. Inhibition of quorum sensing may be beneficial in attenuation of virulence and the treatment of infections caused by resistant strains of bacteria^26^,^27^.

The tripyrrole red prodigiosin pigment is synthesized under the control of quorum sensing in *S. marcescens*^21^. GTN caused remarkable reduction in prodigiosin pigment. Protease is involved in the pathogenicity of *S. marcescens*. GTN reduced the production of protease^28^.

In this study, GTN showed high ability to inhibit biofilm. Biofilms render the infection difficult to treat due to the high resistance to antibiotics and immunity of the host^29^. The ability of *Serratia marcescens* to swim and swarm affects adhesion and biofilm formation. As a result, interference with swarming could affect biofilm production^30^,^31^. GTN blocked swarming motility.

The inhibition of virulence factors production of *Serratia marcescens* may be due to interference with quorum sensing. This hypothesis was proved by the ability of GTN to cause significant reduction in the production of the quorum-sensing regulated *violacein* pigment in the biosensor strain *C. violaceum* CV026. Moreover, GTN was previously reported as a quorum sensing inhibitor in *Pseudomonas aeruginosa* PAO1 strain^11^.

GTN has the advantage of FDA approval that makes GTN clinical application possible. This is very important because most quorum sensing inhibitors have toxic effects that hinder their use in treatment. *Serratia marcescens* causes surgical wounds infections and urinary tract infections. GTN can be used topically in the treatment of surgical site infections or as a catheter lock solutions for catheter associated urinary tract infections^32^.

## Conclusion

The emergence of antibiotic resistance makes it obligatory to seek for new therapeutic options for infection control. GTN may be a useful agent in this approach because of its anti-quorum sensing and anti-virulence activities against *S. marcescens*. Targeting quorum sensing regulated virulence poses no growth pressure on bacteria and hence the resistance is much less likely to develop.
